# Healthcare cost and utilization for chimeric antigen receptor (CAR) T‐cell therapy in the treatment of pediatric acute lymphoblastic leukemia: A commercial insurance claims database analysis

**DOI:** 10.1002/cnr2.1980

**Published:** 2024-01-13

**Authors:** Alex Hoover, Paige Reimche, Dave Watson, Lynn Tanner, Laura Gilchrist, Mike Finch, Yoav H. Messinger, Lucie M. Turcotte

**Affiliations:** ^1^ Optum Labs Visiting Fellow, Department of Pediatrics University of Minnesota Minneapolis Minnesota USA; ^2^ Department of Pediatrics Children's Minnesota Minneapolis Minnesota USA; ^3^ Graduate College, Division of Physical Therapy St. Catherine University St. Paul Minnesota USA

**Keywords:** ALL, CAR‐T, cost, pediatric

## Abstract

**Background:**

B‐lineage acute lymphoblastic leukemia (B‐ALL) is the most common malignancy of childhood. With the introduction of novel cellular therapies, cost of care is a critical component and the financial burden experienced by patients and society requires evaluation.

**Aims:**

This study aims to assess the utilization and cost of care for chimeric antigen receptor T‐cell (CAR‐T) therapy for pediatric ALL patients with commercial insurance coverage in the United States.

**Methods and Results:**

Using de‐identified commercial insurance data from the OptumLabs® Data Warehouse, a cohort of 37 patients, aged 1‐25 years, with B‐ALL treated with CAR‐T therapy between Oct 2016 and Dec 2021 in the United States was identified. Cost was evaluated for a 90 day period encompassing CAR‐T infusion and by administration and complication characteristics. Among the 37 identified B‐ALL patients that received a CAR‐T product infusion, 14 patients were female, median age at administration was 13 years. The median 90‐day total cost was $620,500 (Mean: $589,108). Inpatient cost accounted for approximately 71% of the total cost with an average of 28 inpatient days per patient. Although inpatient cost was slightly higher in the older age group (aged 10‐25 years) and in patients with a code for cytokine release syndrome (CRS), these differences were not statistically significant.

**Conclusion:**

This real‐world cost analysis shows for the first time the encompassing cost of CAR‐T therapy for pediatric B‐ALL patients in the US with commercial insurance. This study provides a valuable benchmark that can be used to analyze the financial implications of CAR‐T therapy for pediatric B‐ALL therapy on health systems.

## 1 INTRODUCTION

Acute lymphoblastic leukemia (ALL) is the most common cancer of childhood and adolescence, with approximately 3000 new cases diagnosed each year in individuals under age 18 years of age in the United States (US).^1^ Approximately 85% of pediatric ALL expresses B‐cell lineage cell‐surface and cytoplasmic markers (B‐ALL).^2^ Over the last five decades, survival of children with B‐ALL has improved drastically from less than 10% in the 1960s to over 90% today.^2^, ^3^ However, relapsed and refractory B‐ALL remains a significant and challenging issue with 5‐year survival for patients with relapsed disease ranging from 40% to 60%.^4^, ^5^, ^6^, ^7^

Chimeric antigen receptor (CAR) T‐cells targeting CD19 on B‐ALL leukemic blasts have changed the landscape of treatment for relapsed and refractory disease. The landmark ELIANA trial that led to the US Food and Drug Administration (FDA) approval of Novartis's tisagenlecleucel (Kymriah), a CD19 CAR‐T product, showed an 82% rate of complete remission (CR) or CR with incomplete hematologic recovery (CRi) in 79 infused children, adolescents, and young adults.^8^, ^9^ Among 65 responders, the median relapse‐free survival (RFS) was 43 months and 5‐year RFS rate was 44% (95% confidence interval (CI), 31%–56%).^10^ In addition to prompting FDA approval for tisagenlecleucel, this trial and subsequent studies of the real world efficacy of this therapy have shown that CAR‐T therapy can be a curative treatment option for heavily pretreated pediatric and young adult patients with relapsed or refractory B‐ALL.^11^, ^12^, ^13^, ^14^

Upon FDA approval for tisagenlecleucel in 2017 for patients up to 25 years of age with B‐cell precursor ALL that is refractory or in second or later relapse, the product was priced at $475 000 for commercial use in the United States.^15^ Notably however, this price tag does not capture the cost to payers for the full course of treatment for patients who receive this therapy. Total cost of the therapy includes that of leukapheresis, lymphodepleting chemotherapy, treatment of complications such as cytokine release syndrome (CRS) and immune effector cell‐associated neurotoxicity syndrome (ICANS) and hospitalization for necessary supportive care.^16^ In the ELIANA trial, 77% of patients experienced some degree of CRS and 48% received tocilizumab,^8^ an IL‐6 monoclonal antibody that also contributes to the total cost of care outside the listed price of tisagenlecleucel.^17^

There are limited data regarding the comprehensive cost of delivering commercial cellular therapy in the US. Previous studies have been limited to cost‐effectiveness analyses based on listed drug prices or have used algorithms or proxies as estimates for cost.^17^, ^18^, ^19^, ^20^, ^21^ There are also multiple studies investigating the cost of CAR‐T therapy delivery in other countries' health systems,^22^, ^23^, ^24^, ^25^, ^26^, ^27^, ^28^, ^29^ however studies of comprehensive real‐life cost of cellular therapy for pediatric B‐ALL are not available in the US.

Previously, our group utilized commercial insurance claims data to investigate the utilization and cost of pediatric ALL care in newly diagnosed patients in the US.^30^ With rising US healthcare costs and the profound financial burden of childhood cancer to patients and society,^31^ it is imperative that we understand the current cost of treatment so that the incremental cost and benefit of future therapeutic modifications can be considered. The aim of this study was to use administrative claims data to evaluate the real‐world comprehensive cost of CAR‐T therapy for B‐ALL in the US, for a cohort of commercially insured pediatric patients.

## 2 MATERIALS AND METHODS

### 2.1 Data Source

OptumLabs® Data Warehouse includes de‐identified medical and pharmacy claims for over 88 million commercially insured individuals living in the US.^32^ The database contains de‐identified, longitudinal health information on enrollees and patients, representing a mixture of ages, ethnicities, and geographical regions across the US. A broad range of clinical and demographic data are available, including reimbursement and clinical utilization. Publicly insured patients and those receiving care through managed‐care organizations are not included in the database. This study was reviewed by the institutional review board at the University of Minnesota.

### 2.2 Cohort identification and outcomes

A cohort of patients aged 1–25 years, with ALL who received CAR‐T therapy between Oct 2016 and Dec 2021 in the United States was identified. CAR‐T product administration was confirmed with use of International statistical Classification of Diseases (ICD‐10) codes for non‐specific or specific CAR‐T products: XW033C3, XW033C7, XW033J7, XW043C3, XW043C7, XW043J7; or the Current Procedural Terminology (CPT) code for the administration itself: 0540T. ALL diagnosis was confirmed based on ICD‐10 diagnostic codes: C91.00, C91.01, C91.02. To be included, individuals were required to have codes for both ALL diagnosis and CAR‐T product administration, as well as continuous insurance coverage for 30 days prior to and 60 days after CAR‐T product administration, unless patient death occurred during this time period. This time frame was chosen to include the majority of therapy‐related complications while avoiding variation from pre‐leukapheresis salvage therapies and potential post‐infusion relapse therapies.

Outcomes of interest included both cost and utilization of care. Here, cost is defined as total reimbursement paid by the commercial payer. Inpatient cost corresponds to claims with a place of service at an inpatient hospital, and outpatient cost correspond to a place of service at an office or hospital‐based outpatient clinic. The reimbursement for care provided during this period can be affected by a combination of care reimbursement negotiation with the payer and the distinct labeling of payments. Therefore, not all of the care provided in the inpatient setting is necessarily paid within the reimbursement that has been labeled “inpatient” as some pharmacy and other charges may be labeled “outpatient” however this should be consistent across the dataset. Utilization was summarized as inpatient days, inpatient encounters, and outpatient encounters. An inpatient encounter corresponded to claims with an inpatient place of service with distinct start and end dates, and an outpatient encounter was defined as a unique physician claim with the place of service either being an office or hospital‐based outpatient clinic.

Prior to October 2020, ICD‐10 diagnosis codes for CRS or ICANS had not been adopted by the Centers for Medicare & Medicaid Services. This prevents the identification of these diagnoses prior to this timepoint without use of unvalidated methods and therefore evaluations of these complications are somewhat limited.

### 2.3 Statistical analysis

Total cost and utilization were summarized for the cohort for the 90‐day time period of interest. Cost was further characterized by the location of service provided, the presence of ICD‐10 codes for the complications of CRS (D89.831‐D89.835, D89.839) and/or ICANS (G92.00‐G92.05). Total cost was analyzed by age group (1–9 years vs. 10–25 years) as a proxy for upfront ALL risk group and by the occurrence of CRS. Analyses were performed using R.^33^

## 3 RESULTS

A cohort of 37 patients aged 1–25 years was identified with a concomitant ICD‐10 code for ALL and CPT code for CAR‐T cell administration (Table 1). All patients had continuous coverage enrollment from 1 month prior to infusion through 2 months post‐infusion except patients who died during the time period. Thirteen patients (35%) were under 10 years of age at the time of cell infusion; median age at administration was 13 years (interquartile range [IQR] 7–19 years). Fourteen patients (37.8%) were female, and while 14 patients were reported to be White, race and ethnicity were unknown in 43% of individuals.

**TABLE 1:** Demographics.

Characteristic	Category	*N* (%)—37
Age	0–9	13 (35)
	10+	24 (65)
Sex	Female	14 (38)
	Male	23 (62)
Setting of CAR‐T therapy	Inpatient	>70%
	Outpatient	<30%
Region	Midwest	–
	Northeast	–
	South	14
	West	11
	Unknown	–

*Note*: Blank cells correspond to censoring cells with less than 11 counts; multiple cells need to be blanked (even if not <11) and percentages adjusted so frequencies cannot be recovered, per OptumLabs guidelines.

**FIGURE 1:**
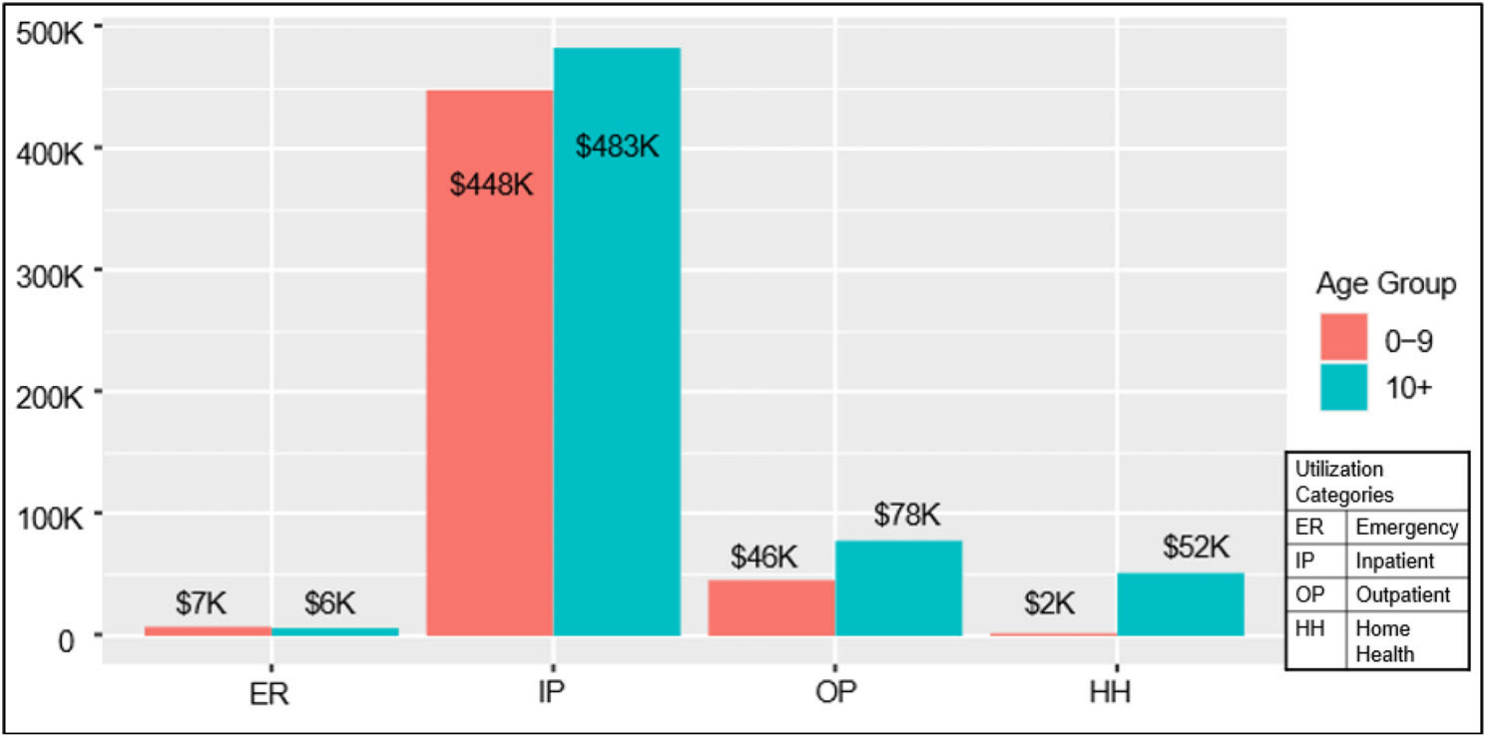
Mean cost by service location & age.

Over the 90 day period encompassing 30 days prior to CAR‐T infusion through 60 days post‐infusion, the median cost for the full cohort was $620 500 (mean, $585398). Overall utilization and cost findings are shown in Table 2. Median cost was not significantly different between patients under vs. over age 10 ($620 279 vs. $633 137, respectively). Inpatient cost accounted for approximately 71% of the total cost with a median inpatient cost of $556 492. Median outpatient cost was $70 545. There were no statistically significant differences between inpatient or outpatient total cost between the two age cohorts (Figure 1).

**TABLE 2:** Analysis of median and mean total care utilization and cost.

		90 day analysis period				
Utilization		Median	Mean	Q1	Q3	*p*‐Value
Inpatient encounters	Overall	2.00	1.88	1.00	2.00	
	0–9 years	2.00	2.33			.1299
	10+ years	1.00	1.62			
Inpatient days	Overall	21.00	28.15	10.00	27.00	
	0–9 years	20.50	26.17			.7307
	10+ years	21.00	29.29			
Outpatient encounters	Overall	17.00	16.78	12.00	22.00	
	0–9 years	19.00	19.38			<.001
	10+ years	16.00	15.38			
	Before CAR‐T	7.00	7.38	5.00	10.00	
	After CAR‐T	9.00	8.81	6.00	12.00	

Cost		Median ($)	Mean ($)	Q1 ($)	Q3 ($)	
Total cost	Overall	$620500.40	$589108.20	$299922	$668395	
	0–9 years	$620279.20	$553328.10			.6287
	10+ years	$633137.20	$608489.10			
Inpatient cost	Overall	$556492.04	$465191.20	$120000	$620690	
	0–9 years	$565398.00	$451359.00			.8225
	10+ years	$234144.00	$472408.00			
Outpatient cost	Overall	$70545.08	$148253.40	$33488	$108938	
	0–9 years	$38311.87	$133205.61			.7426
	10+ years	$80038.59	$156758.66			

The median number of inpatient encounters across the cohort was 2, with patients spending a median of 21 days in the hospital. Patients had an average of 7 outpatient visits in the 30 days prior to CAR‐T therapy and an average of 9 outpatient visits in the 60 days following infusion. Similar to cost, there was no significant difference between the utilization of care based on age.

**FIGURE 2:**
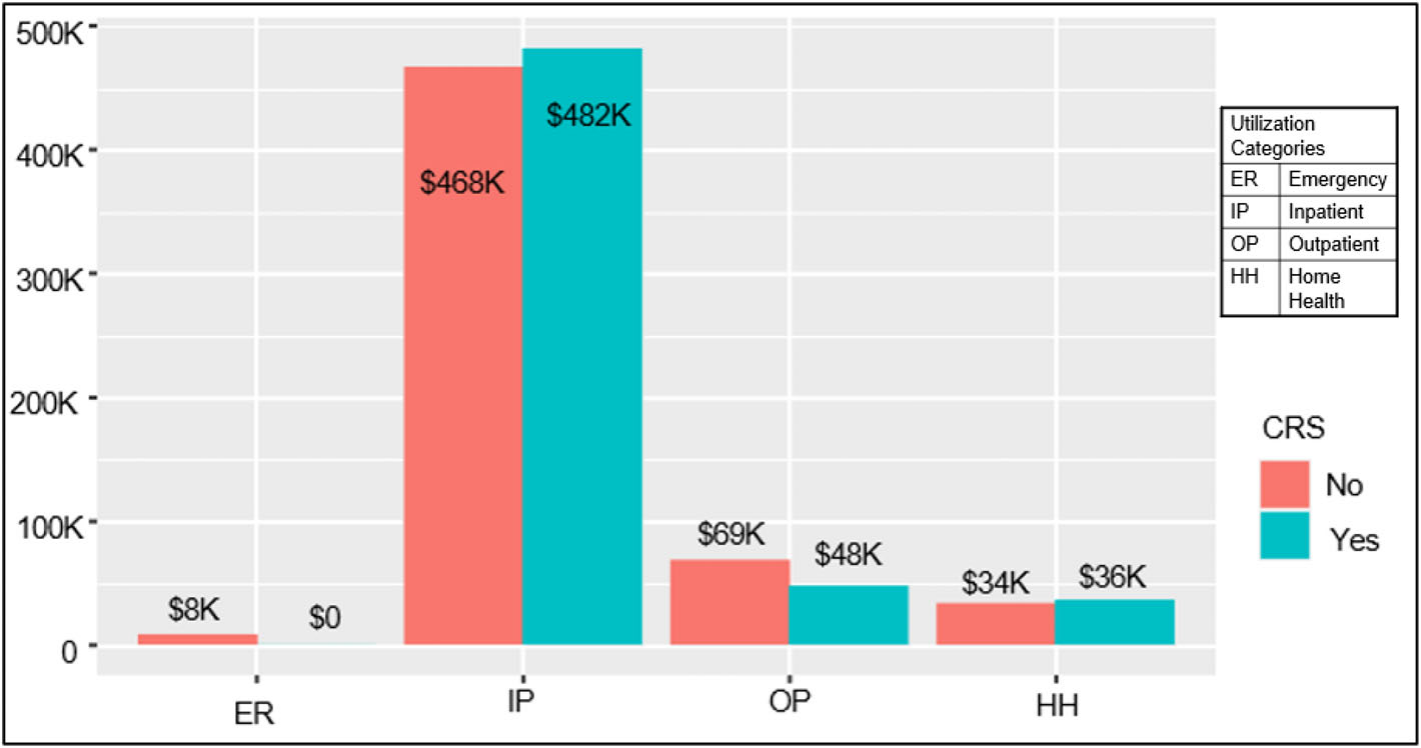
Mean cost by service location & cytokine release syndrome occurrence.

Within the cohort, less than 11 patients had a diagnostic code for cytokine release syndrome at some point during the study period (*n* < 11 cannot be identified per OptumLabs data guidelines). No patients in the dataset had a diagnostic code for ICANS. When stratified into CRS and non‐CRS cohorts, there were no significant differences in total, inpatient or outpatient cost between the cohorts (Figure 2).

Additionally, no patients in the cohort had a CPT code for allogeneic hematopoietic stem cell transplant (alloHCT) during the 60‐day period post‐infusion of CAR‐T cells.

## 4 DISCUSSION

In this study, we examined the healthcare cost and utilization for pediatric patients receiving CAR‐T cell therapy for B‐ALL using a commercial claims database, the first analysis of its kind in this patient population. Patients were predominantly male and most were adolescents, which is consistent with prior studies that have shown that in the pediatric population, male sex and age > 10 years are significant predictors of inferior post‐relapse survival and therefore require novel therapies such as CAR‐T.^34^

Almost all patients received the CAR‐T therapy in the inpatient setting, and thus, inpatient cost was significantly higher than outpatient cost, likely reflecting the price of the tisagenlecleucel product itself in addition to the cost of lymphodepleting chemotherapy and care for post‐infusion complications. Costs of emergency department care and home health‐related care contributed minimally to the overall cost burden in the 90 day period of interest around CAR‐T infusion.

The median total cost across the entire cohort for the period of interest was $620 500, indicating that the typical cost of care, excluding the listed drug price for tisagenlecleucel of $475 000, was approximately $145 500. This is fairly consistent with cost modeling performed for tisagenlecleucel in pediatric B‐ALL patients by Lin et al. which predicted the total cost to be between $548 000 and $599000.^18^ This is also consistent with other reports of cost associated with the real‐world care for patients receiving CAR‐T therapies in adults with relapsed or refractory lymphomas.^35^, ^36^

Our study has several limitations. The landmark ELIANA trial of tisagenlecleucel study reported a CRS rate of 77% and neurotoxicity rate of 40% with this therapy.^8^ Prior to October 2020, no ICD‐10 diagnosis codes for CRS or ICANS had been adopted by the Centers for Medicare & Medicaid Services. Therefore, other studies have utilized claims‐based algorithms for CRS and neurotoxicity via expert clinical opinion and based on the clinical manifestations of these complications.^35^ However, given the lack of ability to validate these algorithms with electronic medical record (EMR)‐based records, the interpretation of these algorithms is challenging. Less than 11 patients in our cohort had a recorded ICD‐10 for cytokine release syndrome (CRS) and no patients had a diagnostic code for immune‐effector cell associated neurotoxicity syndrome (ICANS). This is expected given the cohort was primarily treated prior to October 2020 when ICD‐10 codes for these diagnoses were introduced, however it unfortunately impacts our ability to detect whether there was higher cost and utilization of care for patients with versus without CRS. Cost that may not have been captured in this analysis include those of leukapheresis, which often occurs greater than 30 days prior to the administration of the CAR‐T product, as well as the longer term cost of B‐cell aplasia and secondary hypogammaglobulinemia following CAR‐T infusion, treated with immune globulin replacement therapy.

Consistent with most commercial claims databases, some demographic and clinical characteristics, such as race/ethnicity or prior therapies such as stem cell transplant, were absent or missing from the OptumLabs data.^35^ This limits our ability to analyze the effect of race/ethnicity on cost, a key component of health equity and opportunity in the current era of healthcare in the United States. The relatively small sample size of this study limits the power to detect differences between cohorts and the lack of clinical information or ability to validate diagnostic coding with EMR‐based data limits analysis of the effect of disease characteristics, performance index or prior therapies and hinders cost–benefit analyses. The same limitations apply to the identification of relapsed/refractory disease post‐infusion, and therefore we are unable to report response outcomes or correlate outcomes with cost or utilization. Additionally, the OptumLabs Data Warehouse is limited to commercially insured individuals and does not account for out‐of‐pocket payments, thus excluding publicly insured or managed care patients from this analysis and limiting the generalizability of these results.

An important consideration regarding the context for this study is the cost of alternative therapies, which in the setting of relapsed/refractory B‐ALL, include either salvage chemotherapy or immunotherapy and/or alloHCT. In the current era, only CAR‐T cell therapy or alloHCT would be considered potentially curative therapies for these patients. A recent investigation of the total cost of alloHCT for pediatric malignancies reported a median total cost of $405810 (range: $178900–$1163667).^37^ Although reporting the cost of alloHCT in the OptumLabs dataset is outside the scope of the current study, considering the context of alternative therapy options and their cost is imperative.

## 5 CONCLUSIONS

This robust real‐world cost analysis shows for the first time the true cost and peri‐infusion care utilization of CAR‐T therapy for pediatric B‐ALL. This encompasses not only the commercial cost of the cellular therapy product itself, but the care involved prior to cell product infusion and the management of its complications. The total cost and utilization of care is not significantly impacted by patient age. Mean and median total cost well exceeded $500000 US dollars, as expected with the listed commercial price of tisagenlecleucel of $475 000. This study provides a valuable benchmark that can be used to analyze the financial burden of CAR‐T therapy for pediatric ALL therapy on health systems. The cost of this therapy can be reassessed over time as other novel therapeutics are introduced into ALL therapy and long‐term outcome data for this therapy are established.

## PRIOR PRESENTATIONS

Results from this study were presented at the 2022 American Society of Hematology (ASH) Annual Meeting, New Orleans, LA, 12/11/2022.

## DISCLAIMERS

The content is solely the responsibility of the authors and does not necessarily represent the official views of the National Institutes of Health or the U.S. government.

## AUTHOR CONTRIBUTIONS

**Alex Hoover:** Conceptualization (lead); investigation (lead); methodology (equal); supervision (equal); writing – original draft (lead); writing – review and editing (lead). **Paige Reimche:** Data curation (lead); formal analysis (lead); methodology (equal); software (equal); visualization (lead); writing – original draft (equal); writing – review and editing (equal). **Dave Watson:** Data curation (equal); formal analysis (equal); investigation (equal); methodology (equal); writing – original draft (equal); writing – review and editing (equal). **Lynn Tanner:** Formal analysis (equal); investigation (equal); writing – original draft (equal); writing – review and editing (equal). **Laura Gilchrist:** Formal analysis (equal); investigation (equal); writing – original draft (equal); writing – review and editing (equal). **Mike Finch:** Data curation (equal); formal analysis (equal); investigation (equal); methodology (equal); writing – original draft (equal); writing – review and editing (equal). **Yoav H. Messinger:** Formal analysis (equal); investigation (equal); writing – original draft (equal); writing – review and editing (equal). **Lucie M. Turcotte:** Conceptualization (equal); formal analysis (equal); funding acquisition (lead); investigation (equal); methodology (equal); resources (lead); supervision (lead); writing – original draft (equal); writing – review and editing (equal).

## FUNDING INFORMATION

This work was supported in part by the NIH‐NRSA Research Fellowship in Translational & Genomic Pediatric Cancer Epidemiology—T32CA099936 (AH), the National Cancer Institute Grant No. K08CA234232 (LMT); the Pine Tree Apple Classic Fund (LG, YHM); and the Children's Cancer Research Fund (LMT).

## CONFLICT OF INTEREST STATEMENT

The authors have no conflicts of interest to disclose.

## ETHICS STATEMENT

All methods were carried out in accordance with relevant guidelines and regulations. This study was reviewed by the Institutional Review Board of the University of Minnesota on 7/19/2021 and assigned a determination of “Not Human Research” under exemption clause 4: “Collection or study of existing data, documents, records, pathological specimens, or diagnostic specimens if publicly available or information is recorded by investigator in a manner that subjects cannot be identified.” Therefore, the need for informed consent was waived by the IRB of the University of Minnesota.

## Data Availability

The data that support the findings of this study are available from OptumLabs. Restrictions apply to the availability of these data, which were used under license for this study. Data are available upon request from the corresponding author with the permission of OptumLabs.
